# Comparison of *MET* gene amplification analysis by next-generation sequencing and fluorescence *in situ* hybridization

**DOI:** 10.18632/oncotarget.28092

**Published:** 2021-10-26

**Authors:** Christina Schmitt, Anna-Alice Schulz, Ria Winkelmann, Kevin Smith, Peter J. Wild, Melanie Demes

**Affiliations:** ^1^Dr. Senckenberg Institute of Pathology, University Hospital Frankfurt, Frankfurt am Main 60590, Germany; ^2^Wildlab, University Hospital Frankfurt MVZ GmbH, Frankfurt am Main 60590, Germany; ^3^Frankfurt Institute for Advanced Studies (FIAS), Frankfurt am Main 60438, Germany

**Keywords:** next-generation sequencing (NGS), non-small cell lung cancer (NSCLC), fluorescence *in situ* hybridization (FISH), MET amplification, routine diagnostics

## Abstract

*MET* gene alterations are known to be involved in acquired resistance to epidermal growth factor receptor inhibition. *MET* amplifications present a potential therapeutic target in non-small cell lung cancer. Although next-generation sequencing (NGS) and fluorescence *in situ* hybridization (FISH) are conventionally used to assess *MET* amplifications, there are currently no clinically defined cut-off values for NGS, with FISH still being the gold standard. A collective of 20 formalin-fixed paraffin-embedded lung cancer tissue samples (mean age 64 years) were selected based on increased *MET* gene copy number (CNV) status or the presence of mutations detected by NGS (GeneReader, QIAGEN) and were further assessed by FISH (*MET*/CEN7, Zytomed). Of these, 17 tumor samples were *MET*-amplified and one patient was found to have a *MET* rearrangement by NGS, while two samples had no *MET* gene alteration. In contrast to the NGS result, FISH analysis showed only one highly amplified sample and 19 negative samples. The single highly amplified case detected by FISH was also positive by NGS with a fold change (FC) of 3.18 and a mean copy number (CN_MV 10−100%_) of 20.5. Therefore, for the assessment of *MET* amplifications using the QIAGEN NGS workflow, we suggest detecting amplified cases with an FC value of ≥ 3.0 and a CN_MV 10−100%_ value of ≥ 20.0 by FISH. In summary, NGS allows for DNA- and RNA-based analysis of specific *MET* gene amplifications, point mutations or rearrangements.

## INTRODUCTION

The proto-oncogene *MET* encodes for a receptor tyrosine kinase which is a ubiquitously expressed cell surface receptor consisting of an extracellular alpha-chain disulfide-bonded to a membrane spanning beta-chain [1, 2]. The binding of the receptor to its ligand, the extracellular hepatocyte growth factor/scatter factor (HGF), induces dimerization of the receptor and triggers a conformational change [3, 4]. As a consequence, the activity of MET tyrosine kinase is activated and can lead to effects on cell growth, mortality, survival, invasion and angiogenesis [5–7]. Dysregulations in the signaling pathway were first investigated in 1990 as a possible cause of lung cancer [8]. Activation of the MET signaling pathway resulting from *MET* amplification or splice site alterations in *MET* exon 14 is associated with lung cancer growth and metastasis [9, 10]. In addition, *MET* amplification correlate with shorter overall survival in non-small cell lung cancer patients and, therefore, has prognostic value [11–13].

Common alterations are *MET* exon 14 splice site mutations and gene amplifications [14]. *MET* alterations are detected in 3–4% of lung adenocarcinomas and *MET* amplifications in 1–6% [15, 16]. In particular, patients diagnosed with lung adenocarcinoma and *MET* amplification showed acquired resistances when treated with epidermal growth factor receptor (EGFR) inhibitors like Gefitinib or Erlotinib, regardless of *EGFR* mutation status [17, 18]. In one study, resistance to EGFR inhibitors was found in 21% of lung cancer patients. Each of the affected patients displayed a *MET* amplification [19].

For these reasons, MET is considered a potentially targetable oncogenic driver [20, 21]. The tyrosine kinase inhibitors (TKIs) for *MET* amplifications are currently in the clinical stage of approval and show promising therapeutic results. For example, Capmatinib was recently approved by the U.S. Food and Drug Administration (FDA) as the first drug targeting specific mutations in advanced lung cancer [16, 22, 23]. It was developed to interrupt the signaling pathway by selectively binding to MET in order to prevent phosphorylation [24]. In addition, Crizotinib, a multikinase inhibitor used in lung cancer patients with *ALK* or *ROS1* translocations, showed a beneficial effect in patients with *MET* alterations [25, 26].

The detection of *MET* amplification can be performed by several techniques such as polymerase chain reaction (PCR), southern blot, immunohistochemistry (IHC) and fluorescence *in situ* hybridization (FISH) [27–29]. It is very important to reliably detect *MET* changes. The standard analytical method for the detection of *MET* amplifications is FISH. However, in recent years, NGS has become a daily routine in molecular diagnostics and offers new opportunities to analyze *MET* alterations [30]. Despite the important therapeutic significance of *MET* alterations, there are currently no defined cut-off values for NGS.

The goal of this project was to find a way to analyze *MET* amplifications by NGS by defining cut-off values. The results of the NGS pipeline (*n* = 20) were compared to the gold standard FISH.

## RESULTS

### Patient collective

The mean age was 64 years, ranging from 37 to 90 years. The cohort consisted of 10 men (50%) and 10 women (50%). 18 (90%) lung carcinomas and two cancers (10%) of unknown primary were included in the study. All samples were formalin-fixed paraffin embedded (FFPE) tumor tissues.

The patient collective was compiled based on the NGS result, wherein the selection criterion for the samples was the presence of a *MET* amplification and an overall sufficient sequencing quality, based on the quality parameters specified by the analysis software (QCI-A), to avoid false positive cases. Cases without *MET* amplification were also selected as controls. Data from a total of 20 patients were analyzed by NGS. 17 (85%) had a *MET* amplification by NGS and one (5%) a *MET* exon 14 skipping event while two (10%) were classified as wild type (Table 1).

**Table 1:** Characteristics of the patient collective including primary tumor site and NGS results of *MET* amplification according to determination via the nNGM-v1 panel, QIAGEN

Study-ID	Primary tumor site	*MET* Status (NGS)	TC [%]	FC	CN 100%	CN 50%	CN 25%	CN 10%	*p*-value	CN_MV 10–100%_ (NGS)
**1**	Lung	2^a^	40							
**2**	Lung	1	40	1.51	3.02	4.03	6.06	12.16	1.24e-12	6.32
**3**	Lung	1	55	3.18	6.35	10.70	19.41	45.52	0	20.50
**4**	Lung	1	60	2.32	4.64	7.29	12.57	28.44	3.09e-12	13.24
**5**	Lung	1	70	2.49	4.98	7.96	13.92	31.79	4.0e-9	14.66
**6**	Lung	2	40							
**7**	Unknown	1	70	1.48	2.96	3.91	5.82	11.55	2.14e-5	6.06
**8**	Lung	1	50	1.44	2.87	3.74	5.49	10.71	8.69e-5	5.70
**9**	Lung	1	50	1.60	3.20	4.40	6.79	13.98	4.07e-6	7.09
**10**	Lung	1	50	2.20	4.40	6.80	11.60	26.01	0	12.20
**11**	Lung	1	30	1.46	2.92	3.84	5.67	11.18	1.47e-3	5.90
**12b**	Lung	1	80	3.50	6.99	11.98	21.97	51.92	3.18e-6	23.22
**13**	Lung	1	50	1.79	3.58	5.16	8.32	17.79	1.9e-6	8.71
**14**	Unknown	1	60	1.56	3.13	4.26	6.51	13.28	0.01	6.80
**15**	Lung	1	30	1.74	3.48	4.96	7.91	16.78	2.95e-11	8.28
**16**	Lung	1	20	1.51	3.03	4.05	6.10	12.26	1.26e-3	6.36
**17**	Lung	1	60	1.66	3.33	4.65	7.31	15.27	0.01	7.64
**18**	Lung	1	70	1.61	3.23	4.46	6.91	14.28	1.75e-10	7.22
**19**	Lung	1	80	2.61	5.22	8.44	14.88	34.20	5.68e-10	15.69
**20**	Lung	2	40							
**Mean value**				1.86	3.72	5.43	8.87	19.17		
**Max**				3.50	6.99	11.98	21.97	51.92		
**Min**				1.44	2.87	3.74	5.49	10.71		

Showing *MET* status NGS (amplified = 1, wild type = 2), tumor content (TC), fold change (FC), copy number (CN 100%, 50%, 25%, 10%), *p*-value (the closer the value is to 0, the more certain the call) and copy number mean value (CN_MV 10–100%_). Abbreviations: CN: copy number; FC: fold change; *MET*: mesenchymal-epithelial transition; NGS: next-generation sequencing; TC: tumor content. ^a^Not *MET* amplified but presence of a *MET* exon 14 skipping event. ^b^Reduced sequencing quality in the NGS analysis.

All samples were subsequently assessed by FISH in order to proof the amplification status (Table 2).

**Table 2:** FISH results

Study-ID	MV MET/nuclei	MV CEN7/nuclei	Ratio	Result
**1**	2.97	1.88	1.50	Negative
**2**	2.42	2.01	1.20	Negative
**3**	cluster			High-level (not countable)
**4**	3.18	2.97	1.07	Negative
**5**	3.45	3.03	1.14	Negative
**6**	2.03	1.82	1.12	Negative
**7**	2.03	2.02	1.00	Negative
**8**	2.03	1.65	1.23	Negative
**9**	1.95	1.83	1.07	Negative
**10**	3.75	2.32	1.62	Negative
**11**	2.82	2.63	1.07	Negative
**12**	3.45	3.80	0.91	Negative
**13**	2.72	2.65	1.03	Negative
**14**	2.45	2.15	1.14	Negative
**15**	2.25	2.00	1.12	Negative
**16**	1.92	2.05	0.94	Negative
**17**	3.47	3.72	0.93	Negative
**18**	3.88	2.41	1.61	Negative
**19**	2.63	2.15	1.22	Negative
**20**	3.02	2.92	1.03	Negative
**Mean value**	2.76	2.42	1.15	
**Max**	3.88	3.80	1.62	
**Min**	1.92	1.65	0.91	

Abbreviations: CEN7: centromere of chromosome 7; *MET*: mesenchymal-epithelial transition; MV: mean value. Mean value (MV) *MET*/nuclei, CEN7/nuclei, ratio and result.

### *MET* alterations using NGS

QIAGEN calculates the values fold change (FC), copy number (CN) and *p*-value in the analysis of amplifications. These were considered in conjunction with the tumor cell content (TC) of the tissue samples for evaluation. The average FC of the 17 patients that were tested positive for *MET* amplification was 1.86, with a maximum value of 3.5 and a minimum value of 1.44. The copy number (100%) ranges from 6.99 to 2.87 with an average value of 3.72 (Table 1).

### *MET* alterations using FISH

**Figure 1:**
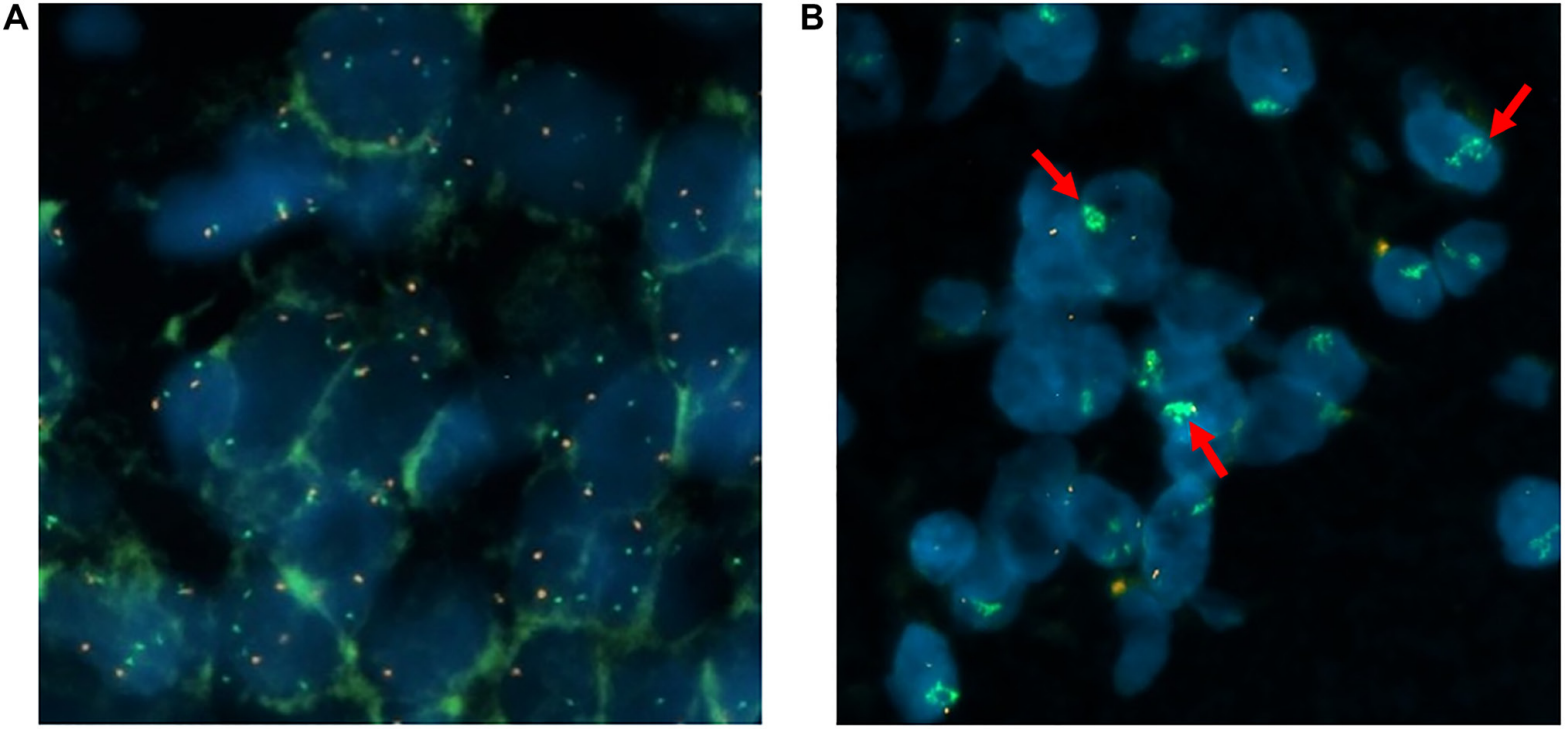
Representation of individual tumor cell nuclei (*MET*/CEN7 probe and DAPI). The *MET* gene is identified with green signals and the centromere of chromosome 7 is the reference gene marked in red: wild type (**A**), cluster amplification (**B**).

The evaluation of the FISH analysis was done by fluorescence microscopy. 20 tumor cell nuclei were counted in three separate areas; i.e., a total of 60 counts per patient. The ratio and mean value per cell (MV) for *MET* and CEN7 were calculated according to the criteria of Schildhaus et al. [31]. The results are shown in Table 2. 19 samples (95%) were negative. One sample (5%) showed a high-level/cluster amplification (Figure 1). No intermediate amplified cases were observed.

### Comparison of NGS (QIAGEN workflow) and FISH analyses

Comparing the results of the two detection methods, the high-level *MET* amplification detected by FISH analysis showed agreement with the result of the NGS analysis. NGS analysis of 20 preselected specimens diagnosed a total of 17 *MET* amplification positive samples and one with a *MET* exon 14 skipping event. Two of the samples showed an FC ≥ 3 (sample 3 and 12). By FISH analysis, a high-level amplification was also detected in sample 3. Sample 12 had a reduced sequencing quality in the NGS analysis, which could be the reason for the discrepancy between the FISH and NGS result. Furthermore, it is not possible to detect exon skipping events by FISH analysis, so the *MET* exon 14 skipping event (^a^) identified by NGS in sample 1 could not be confirmed (Table 3).

**Table 3:** Comparison of NGS and FISH results regarding *MET* amplification

Study-ID	TC [%]	*MET* Amplification Status (FISH)	*MET* Amplification Status (NGS)	FC	CN 100%	CN 50%	CN 25%	CN 10%	CN_MV 10–100%_ (NGS)
**1**	40	2^a^	2^b^						
**2**	40	2	1	1.51	3.02	4.03	6.06	12.16	6.32
**3**	55	1	1	3.18	6.35	10.70	19.41	45.52	20.50
**4**	60	2	1	2.32	4.64	7.29	12.57	28.44	13.24
**5**	70	2	1	2.49	4.98	7.96	13.92	31.79	14.66
**6**	40	2	2						
**7**	70	2	1	1.48	2.96	3.91	5.82	11.55	6.06
**8**	50	2	1	1.44	2.87	3.74	5.49	10.71	5.70
**9**	50	2	1	1.60	3.20	4.40	6.79	13.98	7.09
**10**	50	2	1	2.20	4.40	6.80	11.60	26.01	12.20
**11**	30	2	1	1.46	2.92	3.84	5.67	11.18	5.90
**12**	80	2	1^c^	3.50	6.99	11.98	21.97	51.92	23.22
**13**	50	2	1	1.79	3.58	5.16	8.32	17.79	8.71
**14**	60	2	1	1.56	3.13	4.26	6.51	13.28	6.80
**15**	30	2	1	1.74	3.48	4.96	7.91	16.78	8.28
**16**	20	2	1	1.51	3.03	4.05	6.10	12.26	6.36
**17**	60	2	1	1.66	3.33	4.65	7.31	15.27	7.64
**18**	70	2	1	1.61	3.23	4.46	6.91	14.28	7.22
**19**	80	2	1	2.61	5.22	8.44	14.88	34.20	15.69
**20**	40	2	2						

Abbreviations: CN: copy number; FC: fold change; FISH: fluorescence *in situ* hybridization; *MET*: mesenchymal-epithelial transition, NGS: next-generation sequencing; TC: tumor content. ^a^ *MET* exon 14 skipping event is not detectable by FISH analysis. ^b^Not *MET* amplified but presence of a *MET* exon 14 skipping event. ^c^Reduced sequencing quality in the NGS analysis. Showing tumor content (TC), *MET* amplification status by FISH and NGS (amplified = 1, wild type = 2) fold change (FC), copy number (CN 100%, 50%, 25%, 10%) and copy number mean value (CN_MV 10–100%_).

**Figure 2:**
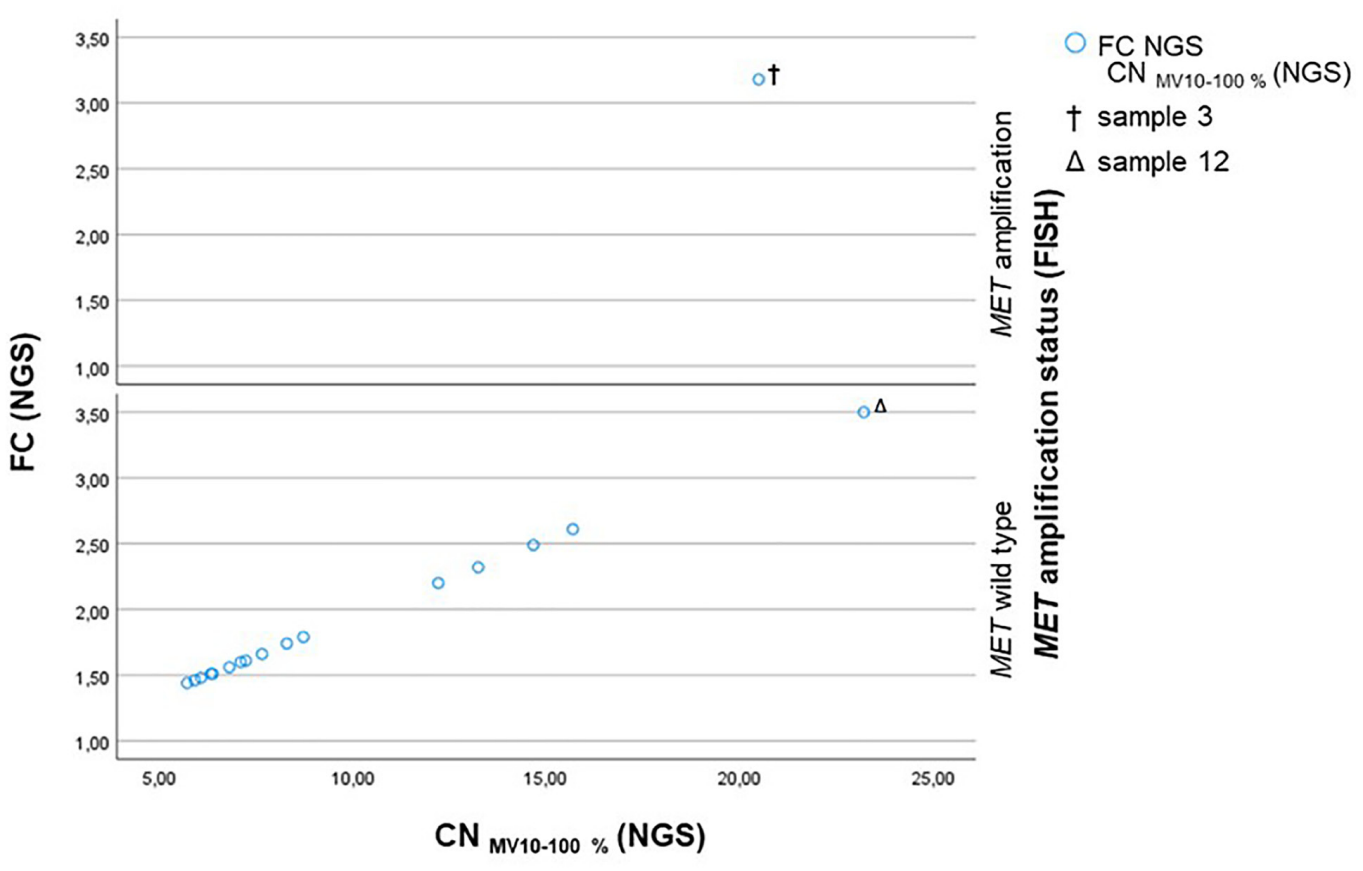
Scatterplot showing the CN_MV 10–100%_ (NGS) plotted against the FC (NGS) subdivided into the *MET* amplification status assessed by FISH. Sample 3 (^†^) is *MET* amplified by FISH and shows a CN_MV 10–100%_ of 20.55 and a FC of 3.18. Sample 12 (^Δ^) is determined as *MET* wild type by FISH and has a CN of 23.22 and a FC of 3.50 due to a low sequencing quality.

All *MET* wild type cases determined by FISH had a FC by NGS ranging from 1.44 to 2.61 and a CN_MV 10–100%_ ranging from 5.70 to 15.69 (Figure 2). With the exception of the outlier sample 12 (*Δ*), which shows a FC of 3.50 and a CN_MV 10–100%_ of 23.22. The only sample (sample 3, ^†^) that was identified as *MET* amplified by FISH presents a FC of 3.18 and a CN_MV 10–100%_ of 20.55 (Figure 2).

### Analytical sensitivity and specificity

For the calculation of the analytical sensitivity and specificity, FISH analysis was assumed to be the gold standard. Thus, considering NGS positive cases with a FC of ≥ 3.0 and a CN_MV 10–100%_ value of ≥ 20.0 as well as sufficient NGS sequencing quality results in both an analytical sensitivity (Equation 1) and an analytical specificity (Equation 2) of 1.

## DISCUSSION

The aim of this study was to verify *MET* amplifications detected by NGS. Therefore, 20 samples with known amplification status (17 amplified cases and 3 non-amplified cases), were additionally checked by FISH.

FISH analysis is the current gold standard and the most widely used method for the detection of increased numbers of gene copies, such as *MET* amplification, with recommended evaluation criteria. For FISH analysis it is recommended to count a total of 50–100 cell nuclei, depending on the present tumor cell content [31]. In this study 60 tumor cell nuclei were counted per sample.

According to Schildhaus et al. FISH analysis enables differentiation between low-level, intermediate-level and high-level amplifications [31], however the therapeutically relevant cut-off value is still a matter of debate [32].

In NGS analysis, low tumor cell content and the sequencing quality influence the amplification results. Thus, false positive or false negative *MET* cases have to be kept in mind. It is not possible to differentiate between artifacts and real gene amplifications via sequencing, as the NGS methodology is only optimized for the detection of fusions and mutations. Amplifications are part of the panel but the sensitivity is limited. The detection of low- or intermediate-level gene amplifications by NGS is still matter of controversy.

In this study no low- or intermediate amplification was observed. Currently, most tumor samples are analyzed by NGS in routine diagnostics. Statistically, only 4% of lung adenocarcinomas show a *MET* amplification detected by FISH and are potential candidates for a targeted therapy [33].

Of the 20 preselected test samples, negative FISH results were consistent with negative NGS results. This fact supports the conclusion that a negative NGS result with a sufficient sequencing quality will strongly predict a negative FISH analysis. Sample 3, which was classified as high-level amplification via NGS (FC 3.18, CN_MV 10–100%_ 20.5, sufficient sequencing quality), showed a positive FISH analysis, too. This therapeutically relevant mutation could be considered for study inclusion and treatment with TKIs. The discrepancy between the results for sample 12 by NGS as compared to FISH can be explained by a decreased NGS sequencing quality. In summary, a cut-off value of ≥ 3.0 and a CN_MV 10–100%_ value of ≥ 20.0 can be recommended in the context of sufficient sequencing quality. This is further supported by the fact that (when positive cases with FC≥3.0 and CN_MV 10–100%_ ≥ 20.0 are considered) an analytical sensitivity and specificity of 1 each can then be calculated, but still some more test samples should be run to finally determine the sensitivity and specificity.

**Figure 3:**
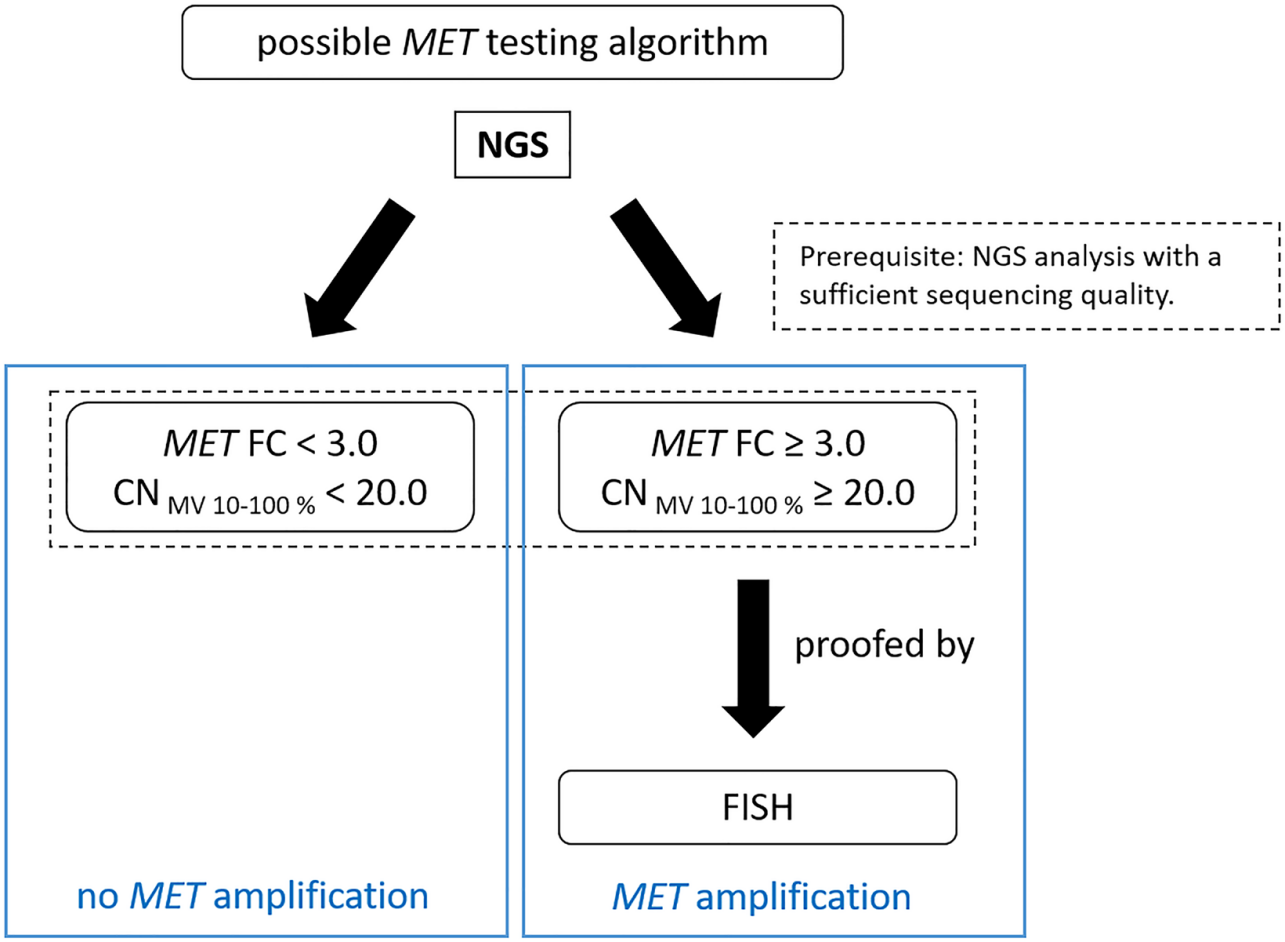
Suggested testing workflow for the detection of *MET* amplifications by QIAGEN workflow.

In conclusion, *MET* alterations appear to be a promising therapeutic target. For this reason, it is important to be able to detect alterations with a high degree of accuracy based on a defined cut-off value. We recommend to use a combination of NGS and FISH for the detection of *MET* amplifications. The exon-14-skipping case (sample 1) in the *MET* gene cannot be detected by FISH, but can be detected by NGS analysis. It can be concluded that amplifications can be verified via FISH sufficiently. With this method each tumor sample can be evaluated microscopically in real time on the basis of the signals per tumor cell but you have to keep in mind overlapping cells to avoid a false increased *MET* signal counting. For NGS, a mixture of tumor, normal, necrotic and inflammatory cells are analyzed, which may reduce validity of the final results. Thus, these methods of molecular pathology complement each other and should be standard in every cancer center. In addition, the cut-off values of the NGS analyses should be specifically defined and validated in each lab depending on the NGS workflow. The following test algorithm is proposed for the detection of *MET* amplifications (Figure 3).

NGS analysis of the samples is performed: If the FC is ≥ 3 and the CN_MV 10–100%_ value is ≥ 20, an additional analysis by FISH should follow to confirm *MET* gene amplification. If the FC is < 3 and the CN_MV 10–100%_ value is < 20, *MET* is considered not amplified. Sufficient sequencing quality is a prerequisite for the NGS based testing algorithm.

Immunohistochemical analysis with recombinant Anti-Met (c-Met) antibody [SP44] – C-terminal (Abcam) as well as a review of the literature did not provide evidence of the validity of MET antibodies for the purposes of prescreening for MET alterations [34, 35].

In the next step we are going to increase the cohort size, including cases with negative, low-level, intermediate-level and high-level *MET* amplification. Additionally, copy number arrays (OncoScan Arrays; Thermo Fisher Scientific) as well as another NGS panel (Oncomine Comprehensive v3; Thermo Fisher Scientific) will be used to validate the results. Further clinical data are needed to define clinically relevant *MET* alterations and to establish a testing algorithm for routine diagnostics.

## MATERIALS AND METHODS

### NGS

The samples were previously analyzed using NGS. After macrodissection of the tumor tissue, DNA was isolated using the Maxwell^®^ RSC FFPE Plus DNA Kit (Promega). The library preparation was performed according to standard laboratory instructions and sequenced using the custom-made targeted resequencing panel (nNGM version 1.0) and the Gene Reader (QIAGEN) workflow. The results were analyzed with the QIAGEN bioinformatics software packages QCI-A and QCI-I. The cases were selected with respect to the quality parameters specified by the analysis software (QCI-A). Sequencing was performed on Gene Reader (QIAGEN) with a resulting sequence coverage of >100×. The detailed parameters of the bioinformatics NGS workflow for the detection of CNVs were set in the QCI-A software according to Table 4.

**Table 4:** CNVs detection configuration (workflow settings configured in the QCI-A software)

Percentile used for fold-change calculation	75
Maximum combined *p*-value for amplification	0.05
Minimum fold change for amplification, absolute value	1.40
Maximum combined *p*-value for deletion	0.05
Minimum fold change for deletion, absolute value	1.40

### FISH

*MET* amplification status was assessed using Zyto*Light*^®^ FISH-Tissue Implementation Kit (ZytoVision^®^) with a dual-color FISH probe set (Zyto*Light*^®^ SPEC *MET*/CEN7*)* targeting *MET* and CEN7 on formalin-fixed, paraffin-embedded tissue sections. The ZytoBrite Hybridizer (Zytomed) was used for hybridization following the Zyto*Light*^®^ protocol. The analysis was made using fluorescence microscopy (Carl Zeiss Microscopy GmbH).

### FISH evaluation

Evaluation of the FISH analysis was performed and modified according to the criteria of Schildhaus et al. [31] (Table 5).

**Table 5:** FISH evaluation sheet modified according to Schildhaus et al. [31]

Not amplified	None of the criteria below is fulfilled
**high-level amplification**	*MET*/CEN7 ratio ≥ 2.0 Average *MET* gene copy number per cell of ≥ 6.0 copies ≥ 10% of tumor cells containing ≥ 15 *MET* signals
**intermediate level amplification**	≥ 50% of cells containing ≥ 5 *MET* signals Criteria for high-level amplification are not fulfilled
**low level amplification**	≥ 40% of tumor cells showing ≥ 4 *MET* signals Criteria for high-level amplification

Abbreviations: CEN7: centromere of chromosome 7; *MET*: mesenchymal-epithelial transition; MV: mean value.

### Calculation of the analytical sensitivity and specificity

To calculate the analytical sensitivity and specificity, FISH analysis was assumed to be the gold standard. The calculation was performed according to the criteria of the “Deutsche Akkreditierungsstelle” (DakkS) [36].

Analytical sensitivity is a measure of the rate of true positive cases. It thus describes the ratio of correctly positive results to the total number of positive results.


$$\begin{array}{cc} sensitivity=\frac{number\;of\;true\;positive}{number\;of\;true\;positive+number\;of\;false\;negative} & \left(1\right) \end{array}$$

The analytical specificity describes the rate of correctly negative results. It is a measure of the ratio of correct negative results to the total number of negative results.


$$\begin{array}{cc} specificity=\frac{number\;of\;true\;negative}{number\;of\;true\;negative+number\;of\;false\;positive} & \left(2\right) \end{array}$$
